# Acetaldehyde involvement in ethanol's postabsortive effects during early ontogeny

**DOI:** 10.3389/fnbeh.2013.00070

**Published:** 2013-06-19

**Authors:** Samanta M. March, P. Abate, Juan C. Molina

**Affiliations:** ^1^Laboratorio de Alcohol, Ontogenia y Desarrollo, Instituto de Investigación Médica Mercedes y Martín FerreyraCórdoba, Argentina; ^2^Department de Psicología, Facultad de Psicología, Universidad Nacional de CórdobaCórdoba, Argentina

**Keywords:** early ethanol exposure, acetaldehyde, ontogeny, learning, appetitive response, suckling, newborns, infants

## Abstract

Clinical and biomedical studies sustains the notion that early ontogeny is a vulnerable window to the impact of alcohol. Experiences with the drug during these stages increase latter disposition to prefer, use or abuse ethanol. This period of enhanced sensitivity to ethanol is accompanied by a high rate of activity in the central catalase system, which metabolizes ethanol in the brain. Acetaldehyde (ACD), the first oxidation product of ethanol, has been found to share many neurobehavioral effects with the drug. Cumulative evidence supports this notion in models employing adults. Nevertheless very few studies have been conducted to analyze the role of ACD in ethanol postabsorptive effects, in newborns or infant rats. In this work we review recent experimental literature that syndicates ACD as a mediator agent of reinforcing aspects of ethanol, during early ontogenetic stages. We also show a meta-analytical correlational approach that proposes how differences in the activity of brain catalase across ontogeny, could be modulating patterns of ethanol consumption.

## Why should we study early ethanol exposure effects?

Epidemiological and preclinical research indicate that prenatal and/or early postnatal ethanol experiences are associated with later responsiveness and affinity to the drug (for review see: Spear and Molina, 2005; Abate et al., 2008; Pautassi et al., 2009). Early initiation in ethanol drinking constitutes a risk factor for the development of later ethanol related problems. Adolescents who begin drinking at age 15 are four-times more likely to become alcohol-dependent than those who start at age 21 (Grant and Dawson, 1997). Ethanol intake usually begins during adolescence, with a decrease in the average age of initiation in the United States from 17.8 years in 1987 to 15.9 years in 1996 (Windle, 2003). Faden (2006) proposed that the peak year for alcohol initiation is even earlier (13–14 years). Heavy drinking in this population is also widespread; with 30% of 12th graders reporting that they had been drunk at least once in the last 30 days (Johnston et al., 2009). Finally, epidemiological data clearly reflects the high prevalence of alcohol use and abuse in both children and adolescents worldwide (e.g., Ahlstrom and Osterberg, 2005). Yet, ethanol exposure can occur involuntarily earlier in ontogeny (Spear and Molina, 2005). In some cultures alcohol soaked gauze pads are employed to avoid infections derived from the remainder of the umbilical cord or to treat stomach spasms (e.g., Dalt et al., 1991; Mancini, 2004). Transdermal absorption of alcohol and inhalation of alcohol vapors can lead to high infantile blood alcohol concentrations (Choonara, 1994). Maternal alcohol drinking during pregnancy can derive in severe damage, such as Fetal Alcohol Syndrome (West, 1994) but also in more subtle effects (i.e., increases the likelihood of ethanol drinking during adolescence: Baer et al., 1998). Ethanol drinking during breastfeeding is still highly prevalent and implies an alternative mode of early exposure to the drug (e.g., Mennella and Beauchamp, 1991; Mennella, 1999; Pepino and Mennella, 2004; Giglia and Binns, 2006). These epidemiological studies highlight the need for developing experimental animal models for understanding the above mentioned effects of early ethanol exposure.

## What do we know about ethanol pharmacological effects in early ontogeny?

Ethanol exerts a wide array of effects. It is rich in calories (7 kcal/g), has a distinctive taste characterized by a combination of sweet and bitter qualities (Molina et al., 2007b). These sensory features can serve as signals conditioned stimulus (CS) that predict biologically relevant events (unconditioned stimulus, US: Molina et al., 1986). Contingent experiences with the scent of alcohol and aversive stimulation result in conditioned avoidance towards the ethanol odor and reduce ethanol intake in 21-day-old rats (Serwatka et al., 1986). Ethanol can also act as an interoceptive context that, when present during the acquisition and retrieval phases of a learning situation, regulates the storage and expression of memories. State dependency mediated by ethanol has been reported in infant, adolescent, and adult rats (Fernandez-Vidal et al., 2003).

Ethanol's sensory features have been proposed to constitute a “taste barrier,” precluding substantial intake of the drug (Pautassi et al., 2008a). Ethanol intake decreases sharply as ethanol concentration increases. If faced with a forced choice between water and a relatively low concentration of ethanol (1–5%), adult heterogeneous rats may show a modest preference for the drug, but ethanol consumption decreases dramatically as higher concentrations are employed (Kiefer et al., 1987; Samson et al., 1988; Kiefer and Morrow, 1991). In contrast, naïve infant rats ingest surprisingly high quantities of ethanol—in concentrations as high as 30%—without initiation procedures (Truxell and Spear, 2004; Sanders and Spear, 2007; Truxell et al., 2007). Early acceptance for highly concentrated ethanol solutions seems to be mediated by the drug's pharmacological properties (Kozlov et al., 2008).

Ethanol induced reinforcement has been documented in neonates and infant rats. Newborn pups rapidly acquire a conditioned response towards an artificial nipple that signals pharmacological effects of very low doses of the drug (Petrov et al., 2003). Ethanol's central injection also promotes positive reinforcement at this age (Nizhnikov et al., 2006c). In infants, first and second order appetitive conditioning has been observed when using low-to high ethanol doses (0.5–2.5 g/kg: Molina et al., 2007a; Pautassi et al., 2008b). Both, locomotor stimulation and reinforcement have been observed during the raising limb of ethanol blood accumulation curve (Petrov et al., 2006; Nizhnikov et al., 2007).

In adult rodents, ethanol induces dopamine release in striatum and nucleus accumbens (Di Chiara and Imperato, 1986; Imperato and Di Chiara, 1986; Di Chiara and Imperato, 1988). The mesolimbic dopaminergic system is involved in ethanol induced motor activity, since D1-like or D2-like receptor antagonists reduce this effect in mice (Pastor et al., 2005). The dopaminergic system seems to modulate ethanol's activating effects also during early ontogeny, with D1 and D2-like receptors antagonist suppressing locomotor stimulation, as shown in adults (Arias et al., 2009b, 2010). However, compared to infants, adult rats seem more prone to ethanol-induced sedation. Locomotor stimulation has been mostly observed in mice or in selectively breed rat strains (Waller et al., 1986; Risinger et al., 1994; Colombo et al., 1998; Agabio et al., 2001). Infant rats show clear biphasic motor effects in response to ethanol, even in response to high doses (2.5 g/kg: Arias et al., 2009a, 2010).

Neurochemical effects of ethanol also include the opiate system as a mediator for DA release (Gianoulakis, 2009; Bodnar, 2012). For example, naltrexone suppresses ethanol self-administration and prevents ethanol-induced increases in dialysate dopamine levels (Gonzales and Weiss, 1998). In relation to early exposure to ethanol, when opiate antagonists are presented with ethanol administration during late gestation, the usual pattern of later enhancement of ethanol ingestion, appetitive orofacial responses and self-administration is prevented (Chotro and Arias, 2003; Arias and Chotro, 2005b; Miranda-Morales et al., 2010). Yet, the opiate system has been found to function differently in neonates compared to adults. Whereas kappa receptor stimulation has aversive effects in adults (Walker and Koob, 2008; Wee and Koob, 2010), newborn rats found it reinforcing (Smotherman and Robinson, 1992, 1994; Nizhnikov et al., 2012). In addition, ethanol reinforcement during this developmental stage requires the joint activation of mu and kappa receptors (Nizhnikov et al., 2006b). During the second postnatal week, a fully functional opioid system is needed to promote ethanol reinforcement. Disruption by either, naloxone or specific opioid antagonists (mu, delta, kappa) is sufficient for substantial reduction in consummatory and seeking behaviors associated with ethanol reinforcement (Miranda-Morales et al., 2012a,b).

Ethanol-derived aversive effects have been easily detected in adult rats, when pairing a taste stimulus (Chester and Cunningham, 1999) or an external context (Philpot et al., 2003) with stages of peak blood ethanol concentrations. In 11-day old infants, an administration of ethanol (3 g/kg) induces conditioned aversions (Molina and Chotro, 1989; Molina et al., 1989). However, the same ethanol dose does not induce conditioned aversion in younger organisms (8 day-old infants: Arias and Chotro, 2006). This developmental switch in ethanol motivational effects is not explained by a deficit in aversive learning capabilities since conditioned aversions are found using lithium chloride as an US (Smotherman, 1982a,b; Miller et al., 1990; Gruest et al., 2004).

The literature reviewed in the present section, along with the biomedical research discussed above, allow us to propose early ontogeny as a sensitive window during which contact with ethanol increases latter disposition to prefer, use or abuse ethanol.

## The acetaldehyde hypothesis

As mentioned, during early ontogeny organisms show a high affinity towards ethanol positive effects, and these early experiences facilitate latter ethanol drinking. Even more, the increasing number of studies showing the role of ethanol metabolites on its postabsortive effects during adulthood (Quertemont et al., 2005), show a profound gap in the literature regarding its effects during early ontogeny; specially when considering that developmental changes in ethanol metabolism have been observed. Following systemic administration of ethanol, higher blood ethanol levels as well as a lower rate of clearance are observed in younger organisms compared to adults (Kelly et al., 1987). Central ethanol metabolism also differs across ontogeny. The catalase system activity, which oxidizes ethanol in the brain, is higher in pups compared to adults (Gill et al., 1992; Hamby-Mason et al., 1997). Thus, ethanol metabolism during early ontogeny seems to derive in high ACD levels in the brain (due to high catalase activity) along with slow ACD formation in the periphery (due to slow EtOH clearance). In addition, aldehyde dehydrogenase –ALDH- activity (acetaldehyde is used as a substrate) in the barrier structures of the brain makes only 10–30% during the antenatal period and increases gradually, reaching the activity specific for mature animals by PD 20–40 (Zimatkin and Lis, 1990).

It is interesting to note that the rate of central/peripheral accumulation of ACD has been involved in the perception of appetitive/aversive effects of the drug. Peripheral accumulation of ACD induces aversive effects (Quertemont, 2004). In fact, aversive reactions induced by ethanol drinking in patients treated with disulfiram [which increases ACD peripheral accumulation and allows peripheral ACD to cross the blood brain barrier, by blocking ALDH activity (Escrig et al., 2012) is the basis of its use in alcoholism's treatment (Kristenson, 1995)]. On the other hand, central ACD formation has been mainly linked to ethanol reinforcing effects (Wall et al., 1992; Hahn et al., 2006). The balance between brain and peripheral formation of ACD can determine the amount of ethanol intake (Chao, 1995). Taking into account these considerations, it is possible to speculate that early ethanol acceptance may be due to high ACD generation in the brain, along with low ACD generation in the periphery.

In spite of cumulative evidence showing that ACD shares most of ethanol effects, such as hypothermic (Closon et al., 2009), locomotive (Correa et al., 2003; Arizzi-Lafrance et al., 2006; Correa et al., 2009; Sanchez-Catalan et al., 2009), sedative (Tambour et al., 2006, 2007), reinforcing (Quertemont and De Witte, 2001; Font et al., 2006a, 2008; Peana et al., 2008), anxiolytic (Correa et al., 2008) and aversive effects (Aragon et al., 1986) in adult rodents, very few studies have analyzed ACD's effects during early ontogeny. In the next section, evidence regarding its involvement in ethanol postabsortive effects in newborn and infant rats will be discussed.

## Behavioral effects of acetaldehyde during early ontogeny

Recently, ACD has been found to have a crucial role in ethanol reinforcement in newborns. Intracisternal administration of ethanol in close temporal contiguity with an odor cue (conditioned stimuli –CS-) derives in increased suckling response to an artificial nipple aromatized with the CS. Yet, this effect is blocked when ACD is inhibited by blocking catalase activity with sodium azide. This effect was specific to ethanol reinforcement since when an alternative central reinforcer was administered (dynorphin), catalase inhibition did not alter subsequent attachment to the scented nipple (Nizhnikov et al., 2007). However, the utilization of catalase inhibitors obstructs certain data interpretation since, along with inhibition of ACD formation, an accumulation of EtOH levels may also take place. Additionally, most catalase inhibitors have unspecific effects such as an impairment of learning produced by sodium azide (Lalonde et al., 1997). Considering this possibility, a study was conducted to corroborate ACD involvement in ethanol reinforcement. Once again, when ACD was neutralized, by inactivating ACD with d-penicillamine, ethanol reinforcement was blocked. Moreover, direct central administration of ACD (0.35 μmol) induced sustained suckling response to an odorized artificial nipple (March et al., 2013a). These studies have been pioneer in showing that ACD has in fact appetitive effects in newborn pups.

**Figure 1:**
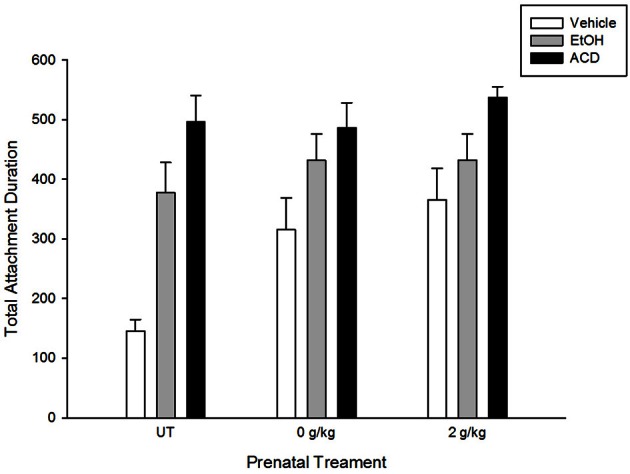
**Total attachment duration in naive newborns (untreated—UT—adapted from March et al., 2013a) and newborns prenatally exposed to water or ethanol (a dayly dose of 2 g/kg during gestational days 17–20; adapted from March et al., 2013b).** Newborns were trained in a clasical conditioning paradigm in which an aromitic cue was associated to a central administration of vehicle, ethanol, or acetaldehyde. Testing took place 1 h later and consisted of presenting an artificial nipple aromatized with the conditioned cue.

ACD reinforcement was also observed by March et al. (2013b), who replicated and extended previous results. Central ethanol or ACD administration induced appetitive conditioning in pups with or without prenatal exposure to ethanol. Pregnant rats received a daily i.g. administration of ethanol (2 g/kg, GDs 17–20). This pattern of prenatal alcohol administration increases appetitive responsiveness to ethanol in newborns (Nizhnikov et al., 2006a; March et al., 2009; Miranda-Morales et al., 2010), infants (Arias and Chotro, 2005a,b), and adolescent rats (Chotro and Arias, 2003). Results showed that ACD induced appetitive conditioning regardless of prior fetal experience with the drug. The conditioned appetitive response to an aromatized artificial nipple was similar to the results reported by March et al., 2013a. The explicit comparison between these studies is represented in Figure 1.

Even though differences in appetitive effects induced by central ethanol (or ACD) administration as a function of prenatal treatments have not been observed, when a higher dose of ACD (0.52 μmol) is centrally administered, motor reactivity is differentially altered. Newborns lacking previous exposure to ethanol showed longer latencies to display motor activity following ACD administration than newborns prenatally exposed to ethanol. Considering this evidence, it is possible that prenatal ethanol exposure may not only induce conditioned appetitive response to the drug's chemosensory properties, but also lead to the development of tolerance to aversive effects induced either by ethanol or its metabolites.

The role of ACD in ethanol's motivational effects were also assessed in 2-week old infant rats (Pautassi et al., 2011). These subjects developed a tactile conditioned preference to a CS (sandpaper) previously paired with ethanol (1 g/kg, i.g.). Conditioning took place during the raising limb of the blood ethanol curve. At this early postadministration time, motor activation was also induced by ethanol. When the authors inactivated ACD by d-penicillamine administration, motivational and locomotive effects of ethanol were inhibited.

Ethanol reinforcing and stimulatory properties appear to be strongly related (Arias et al., 2009a). Interestingly, the ACD dose found to exert appetitive effects in newborns (0.35 μmol) induces motor stimulation in adults (Correa et al., 2003, 2009; Arizzi-Lafrance et al., 2006). The neurochemical bases of these effects have been studied during adulthood. It has been observed that, as well as ethanol, ACD activates the mesolimbic dopamine system (Melis et al., 2007; Diana et al., 2008; Melis et al., 2009). Interestingly, sequestering of central ACD by d-penicillamine prevents ethanol-induced stimulation of the mesolimbic dopamine transmission (Enrico et al., 2009). The opiate system is also involved in ACD reinforcement. Naloxone produced a decrement in schedule-induced ACD self-administration (Myers et al., 1984). Additionally, enhancement of locomotor activity induced by administration of ACD or EtOH into the ventral tegmental area is reduced in animals previously given naltrexone, or β-funaltrexamine (Sanchez-Catalan et al., 2009). The involvement of dopaminergic and opiate activity in ethanol behavioral effects during early ontogeny has been previously discussed. To the extent in which these effects are due to ethanol or to its metabolic products still needs to be determined.

**Figure 2:**
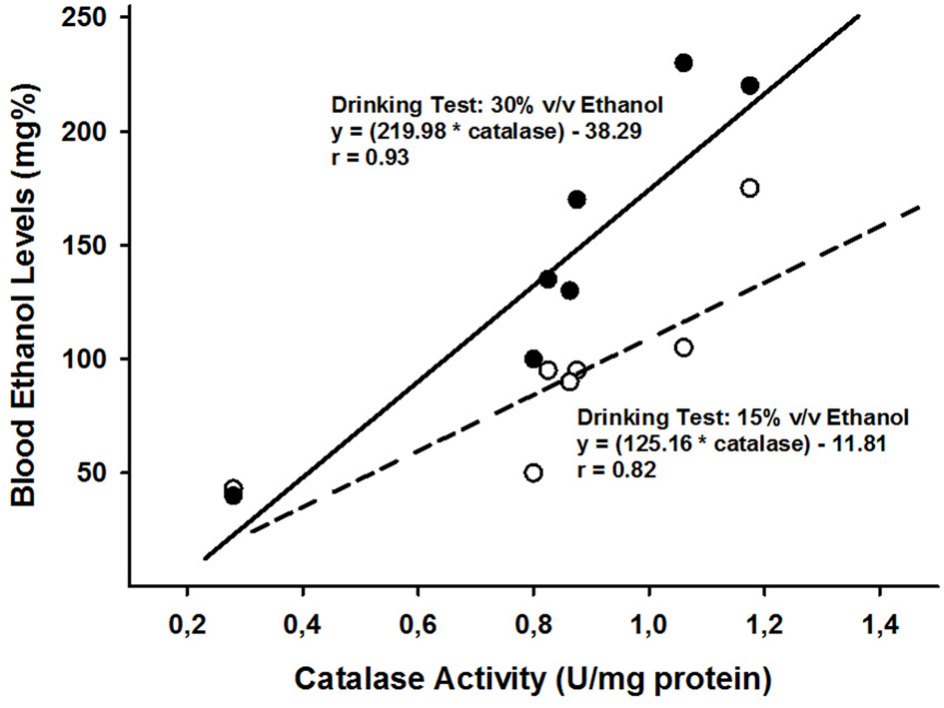
**The figure illustrates regression lines and linear correlations when taking into account catalase activity levels (U/mg protein; data derived from the study of -Del Maestro and Mcdonald, 1987-) and blood ethanol levels attained in alcohol drinking tests performed with either 15 or 30% v/v ethanol (data derived from Truxell and Spear, 2004; Truxell et al., 2007).** In order to perform these correlations, catalase and blood ethanol levels were calculated for the following postnatal days: 12, 18, 22, 25, 28, 30, and 60.

Is there additional support linking early ethanol affinity with high levels of central catalase activity and hence, heightened bioavailability of ACD? Can we find evidence in the literature establishing at least a correlation between levels of catalase activity and ethanol appetitiveness across ontogeny? To our knowledge, not in a specific article. But from a meta analytical perspective, the answer appears positive. Let's explain ourselves in this approach. First, we took into account developmental changes in catalase activity based on average scores (U/mg protein) observed in cerebral hemispheres, striatum, cerebellum and brain stem (Del Maestro and Mcdonald, 1987). There is a gradual decrease in these levels as a function of increasing age. In accordance with the developmental catalase curves shown by these authors, we extrapolated the corresponding values for postnatal days 12, 18, 22, 25, 28, 30, and 60. These values were linearly correlated with those reported by (Truxell and Spear, 2004; Truxell et al., 2007) in terms of blood ethanol levels obtained in alcohol consumption tests, performed at similar ages. Spontaneous ethanol intake also decreases gradually across development. These tests were conducted with relatively high ethanol concentrations (either 15% or 30% v/v ethanol). In both cases the correlations (Pearson's correlation coefficients) were significantly positive. The values of the correlation indexes were as follows: when employing 15% v/v ethanol, *r* = 0.82 (*p* < 0.025) and when utilizing 30% v/v ethanol, *r* = 0.93 (*p* < 0.01). These results have been depicted in Figure 2. From a meta-analytical correlational approach, the hypothesis that differential levels of brain catalase during ontogeny modulates patterns of ethanol affinity, seems to receive support.

Ethanol behavioral and motivational effects (appetitive, aversive and anxyolitic) have been extensively studied in adults and infants. The capability of ACD to induce similar effects has not been comprehensively studied during early ontogeny. For example, negative reinforcement, a property that is believed to play an important role in alcohol use and abuse, has yet to be directly assessed. The gap in contemporary knowledge of the role of ACD in ethanol's postabsortive effects at this developmental period emphasizes the importance of the studies discussed in this section as well as the need for further tests of ontogenetic differences in alcohol acceptance and consumption and the role of EtOH's metabolites in these differences.

## Conclusion

The literature revised here does show that the developing organism can be exposed to ethanol unwillingly. During early ethanol exposure, the organism can learn about ethanol effects (or ACD-mediated effects) and modify its latter responsiveness to the drug (or to associated sensory cues) as a function of these experiences. Compared to the growing body of evidence regarding the modulation of ACD in ethanol effects in adults, little is known about its effects in very young organisms. Even more, to our knowledge, there are none studies addressing acetate effects in newborns or infants.

Until now, ACD levels produced following ethanol administration have not been assessed in newborn or infant rats. The methodological difficulties regarding its measurement *in vivo* and *in vitro* have been discussed elsewhere (Correa et al., 2012) and exceed the purpose of the present review. Yet, studies performed in adult subjects shows compelling evidence signaling ACD's involvement in ethanol effects, since blocking its production (Aragon et al., 1985; Sanchis-Segura et al., 1999; Font et al., 2008; Pastor and Aragon, 2008) or sequestering it (Font et al., 2005, 2006b; Peana et al., 2008; Enrico et al., 2009), inhibits some ethanol behavioral effects. Additionally, potentiating ACD by inducing catalase activity (Correa et al., 1999, 2001) or by administering it directly into the brain (Rodd-Henricks et al., 2002; Correa et al., 2003, 2009; Rodd et al., 2005; Diana et al., 2008; Sanchez-Catalan et al., 2009) mimics the effects typically observed after ethanol administration. Even more, some experimental studies have already started to analyze underlying neurochemical mechanism (Rodd et al., 2003; Hipolito et al., 2009, 2010). We have provided evidence that during early ontogeny ACD has also a role in ethanol reinforcement. Future studies including ontogenetical comparisons are certainly needed.

### Conflict of interest statement

The authors declare that the research was conducted in the absence of any commercial or financial relationships that could be construed as a potential conflict of interest.
